# Significance and challenges of immunopharmacogenomics

**DOI:** 10.3389/fimmu.2025.1711887

**Published:** 2025-12-08

**Authors:** Lara Hanci Handzha, Yusuke Nakamura

**Affiliations:** 1 National Institutes of Biomedical Innovation, Health and Nutrition (NIBN), Ibaraki, Osaka, Japan; 2 Acibadem University Faculty of Medicine, Istanbul, Türkiye

**Keywords:** immunopharmacogenomics, HLA, TCR, BCR, precision medicine

## Abstract

Pharmacogenomics traditionally examines how inherited genetic variations influence drug metabolism, pharmacodynamics, and toxicity. Recent advances have highlighted the immune system as a critical determinant of therapeutic efficacy and safety. Immunopharmacogenomics integrates genetic information, particularly HLA polymorphisms and immune repertoire dynamics of T-cell and B-cell receptors (TCRs and BCRs), to explain interindividual differences in drug responses, immune-related toxicities/diseases. This review summarizes how HLA diversity, immune repertoire heterogeneity, and tolerance mechanisms shape therapeutic outcomes across diverse clinical contexts, including immune-mediated adverse drug reactions, cancer immunotherapy, graft-versus-host disease, autoimmune disorders, food allergy, transplantation, and vaccination. Emerging evidence indicates that immune repertoire sequencing captures dynamic clonal shifts and diversity alterations associated with disease states and treatment responses, providing both mechanistic insight and predictive biomarkers. By integrating genetic and immune repertoire analyses, immunopharmacogenomics establishes a framework for individualized prediction, safer drug design, and more precise immunotherapies, thereby advancing the next phase of precision medicine.

## 1 Introduction

Pharmacogenomics is the study of how an individual’s genetic makeup shapes their response to medications. By integrating pharmacology and genomics, this field seeks to explain why some patients benefit from a drug, while others experience no/limited efficacy or severe adverse effects. Genetic variations contribute to drug response at multiple levels. Inherited germline polymorphisms, including single nucleotide polymorphisms (SNPs), insertions or deletions (indels), and copy number variations (CNVs), can affect drug metabolism, transport, and clearance (1, 2). Variants in drug receptors and downstream signaling pathways may further alter therapeutic efficacy or toxicity. Although pharmacogenomics and pharmacogenetics are sometimes distinguished, the terms are often used interchangeably in practice.

In contrast, immunogenomics focuses on the genetic and genomic determinants of the immune system, which is more complex than the pathways typically studied in classical pharmacogenomics. Most of the literature uses the term “immunogenomics” to describe associations between immune gene polymorphisms, particularly within the human leukocyte antigen (HLA) system, and disease susceptibility. However, the field extends well beyond HLA, encompassing the highly diverse repertoires of T-cell receptors (TCRs) and B-cell receptors (BCRs) that underpin adaptive immune recognition and memory (3). T and B lymphocytes rely on these receptors to distinguish self from non-self and to mediate responses against pathogens, malignant cells, and even drugs, and sometimes against self-cells (3, 4). Because the immune system is both highly dynamic and individualized, elucidating its genomic determinants is indispensable for anticipating, stratifying, and mitigating immune-related drug responses.

Immunopharmacogenomics emerges at the intersection of pharmacogenomics and immunogenomics, aiming to elucidate how genetic diversity in immune-related genes and receptor repertoires and their dynamic changes govern drug efficacy, toxicity, and therapeutic outcomes. Central to this framework are HLA polymorphisms, some of which confer susceptibility to immune-mediated adverse drug reactions (IM-ADRs), exemplified by carbamazepine-induced Stevens–Johnson syndrome or abacavir hypersensitivity. Equally important is the diversity of TCR and BCR repertoires, generated through V(D)J recombination, junctional insertions and deletions, and, in B cells, additional somatic hypermutation (5, 6). The most variable segment, the complementarity-determining region 3 (CDR3), directly contacts antigens and plays a pivotal role in drug-specific immune responses.

Immune receptor repertoires are not static, but highly dynamic, shifting in composition and clonality during infection, disease progression, treatment, and relapse. These changes profoundly influence drug responses, contributing to both therapeutic efficacy (e.g., immunotherapy) and toxicity (e.g., immune-related adverse events). The advent of next-generation sequencing (NGS) transformed repertoire profiling by enabling high-throughput, unbiased characterization of TCR and BCR repertoires at single-base resolution (7, 8). NGS reveals the full landscape of clonal expansions and somatic variations and allows longitudinal tracking of immune dynamics during treatment, relapse, and disease progression, offering both mechanistic and translational insights (8).

The clinical relevance of immunopharmacogenomics is rapidly expanding, reflecting the immune system’s vital role across a wide spectrum of conditions, including infectious diseases, autoimmunity, organ transplantation, food allergies, and drug-induced toxicities (9, 10). In oncology, immune checkpoint inhibitors targeting CTLA-4, PD-1, and PD-L1 have revolutionized therapy, illustrating how modulation of immune pathways can achieve durable tumor control (11). However, these breakthroughs also highlight a critical unmet need for predictive biomarkers: not all patients respond, and some develop severe immune-related toxicities (12). By integrating genetic insights (e.g., HLA variations) with immune repertoire profiling, immunopharmacogenomics offers a framework for individualized prediction of both therapeutic benefit and risk.

**Figure 1:**
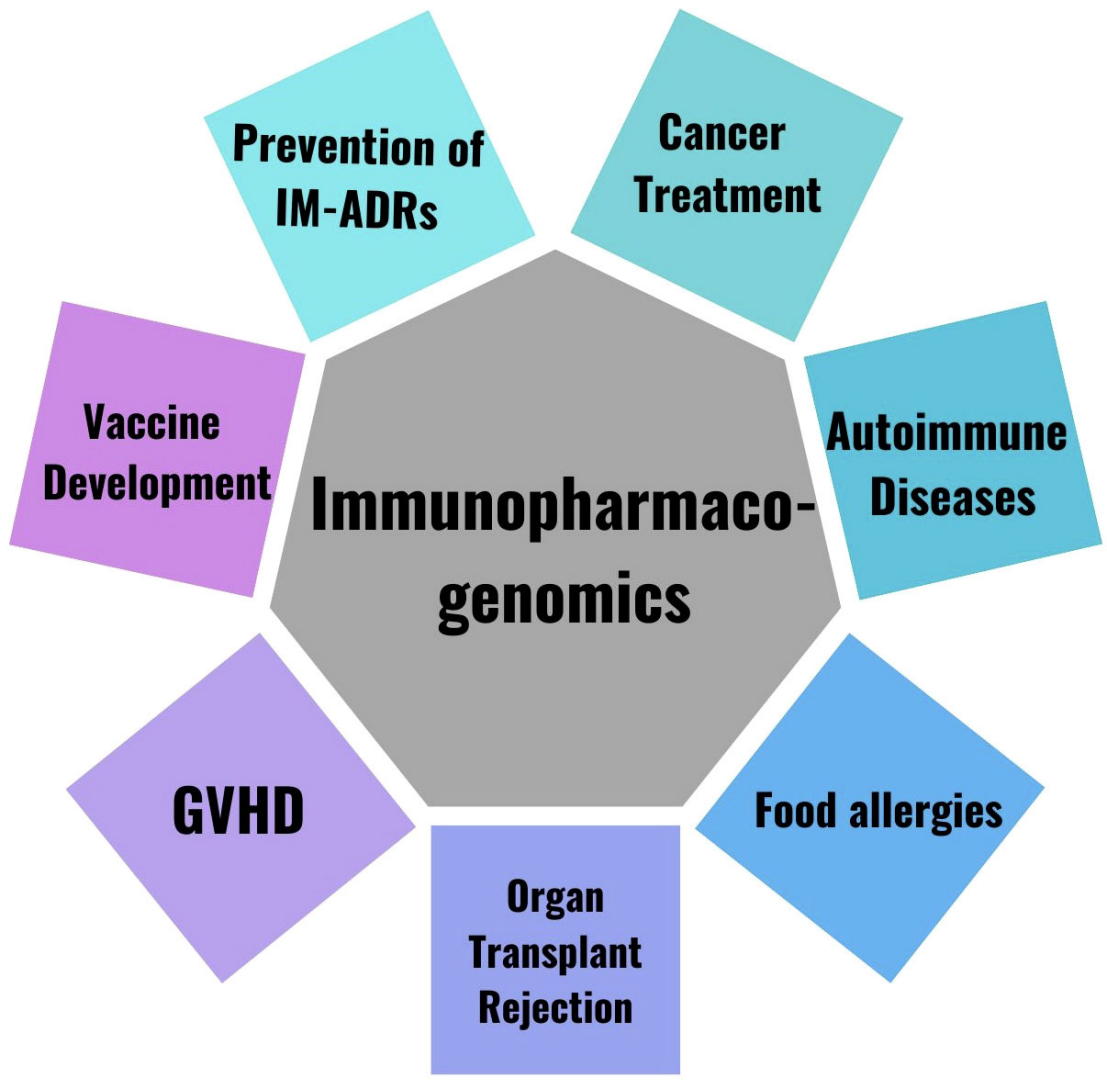
Immunopharmacogenomics is a field that applies genetic insights to immune-related conditions, enabling a better understanding and personalization of treatments across seven key clinical areas shown in this figure. These applications include enhancing “Cancer Treatment” by predicting individual responses and tailoring therapies; investigating “Autoimmune Diseases” and “Food Allergies” by identifying underlying genetic factors; improving outcomes in “Organ Transplant Rejection” and “GVHD (Graft-versus-Host Disease)” through better risk stratification; optimizing “Vaccine Development” for greater efficacy; and predicting and preventing “Immune-Mediated Adverse Drug Reactions (IM-ADRs)”.

In this review, we discuss the mechanisms of immune recognition, the methodologies used to characterize them, and their implications for advancing precision medicine. **Figure 1** presents an overview of major clinical domains where immunopharmacogenomics can have significant impact.

## 2 Fundamental mechanisms of immunogenomics

The fundamental mechanism of adaptive immunity is driven by the interplay of HLA, TCR, and BCR molecules (13). The immune response begins with antigen-presenting cells (APCs) that break down foreign invaders (bacteria, viruses etc.) into small peptides. These peptides are then loaded onto human leukocyte antigen (HLA) molecules and displayed on the cell surface. This HLA-peptide complex is recognized by the highly specific T Cell Receptor (TCR) on lymphocytes. The class of HLA molecule determines which cell subset is activated: HLA Class I molecules present peptides to CD8^+^ cytotoxic T cells, which then kill infected or mutated cells, while HLA Class II molecules present peptides to CD4^+^ helper T cells, which coordinate the broader immune response. Simultaneously, B Cell Receptors (BCRs), which are membrane-bound antibodies, bind to intact, unprocessed antigens on pathogens or circulating in extracellular fluids. Once activated by antigen and CD4+ helper T cell signals, B cells proliferate and differentiate into plasma cells, producing soluble antibodies that neutralize the pathogen or tag it for destruction. The enormous diversity required for this system to recognize virtually any pathogen or cell is generated by the genetic process of V(D)J recombination that creates the huge variations of TCR and BCR repertoires in each individual. The unique combination of an individual’s polymorphic HLA genes and diverse TCR/BCR repertoires shapes the specificity and magnitude of their immune responses, a variability evident in contexts ranging from highly specific drug hypersensitivities to the broader, polyclonal patterns observed in autoimmune diseases and the tumor microenvironment.

## 3 Immune phenotype measurement using repertoire metrics

**Figure 2:**
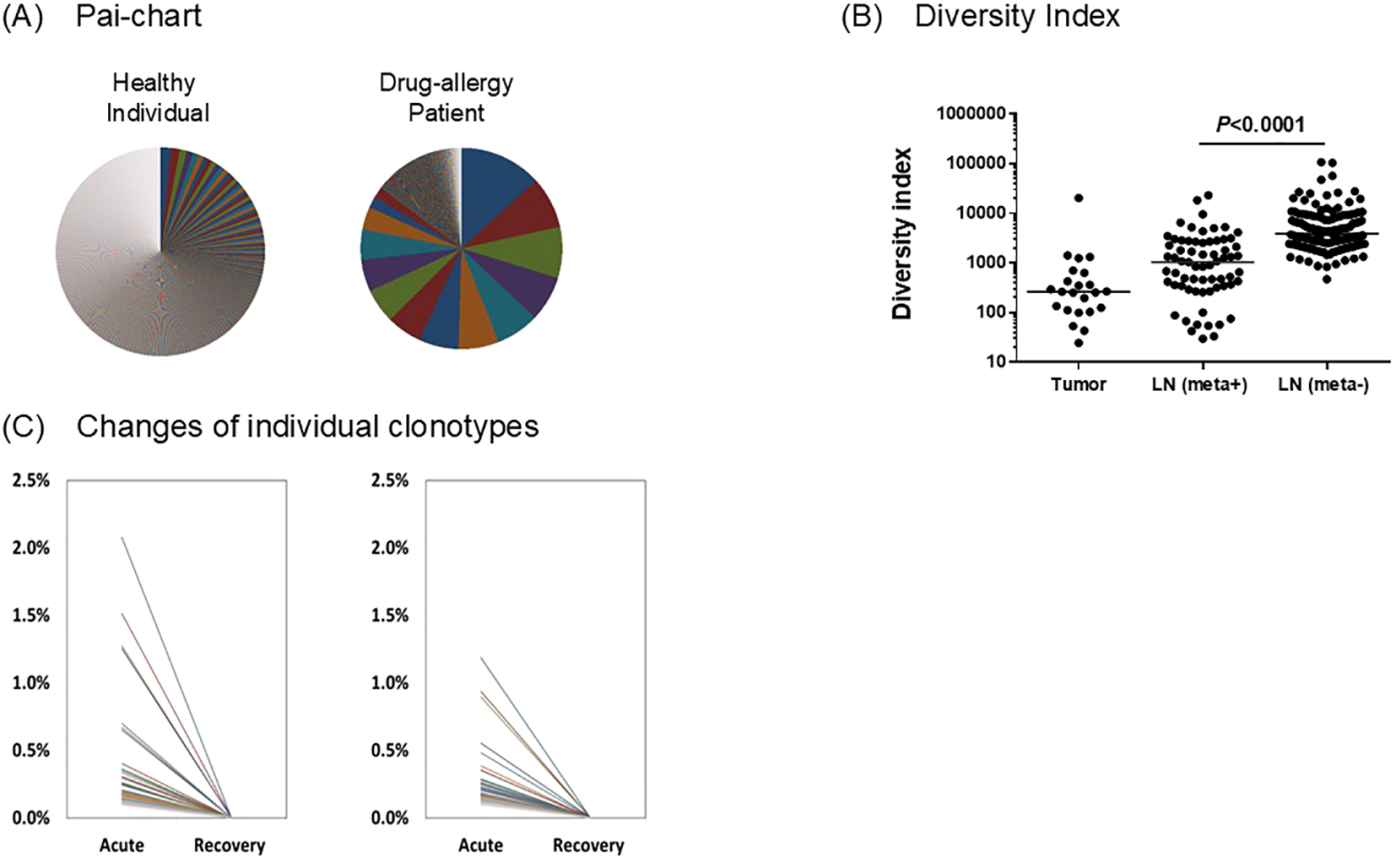
Immune Phenotype Measurement Using Repertoire Metrics **(A)** Comparison of T-Cell Receptor (TCR) repertoire diversity in healthy and drug-induced skin rash individuals shown by pai-charts. (unpublished data) **(B)** displays a gradient of T-Cell Receptor (TCR) diversity across samples from 23 colorectal cancer patients, measured using the inverse Simpson’s diversity index, where higher values denote greater diversity. Diversity is lowest in the primary tumors, indicating a constrained T-cell population, then significantly increases in metastasis-positive lymph nodes (LN (meta+), suggesting a broader immune response recruitment. The highest diversity is observed in the metastasis-negative lymph nodes (LN (meta-), which is significantly higher than in LN (meta+) (P < 0.0001) (13). **(C)** presents the kinetics of clonally expanded B cells in two representative cases of Kawasaki disease (KD), tracking their frequency in the blood between the acute phase and the recovery phase following intravenous immunoglobulin (IVIG) treatment (14).

The adaptive immune system relies on diverse populations of T and B lymphocytes, each expressing a unique receptor created through V(D)J recombination. TCRs and BCRs define clonotypes, or lineages of lymphocytes sharing identical receptor sequences. High-throughput sequencing of these receptors (using tools such as IMREP, IgBLAST, MiXCR, or TRUST4) (14–16) provides a snapshot of the immune repertoire, allowing us to measure and visualize immune phenotypes using pie charts, diversity indices, and clonotype frequency dynamics (**Figure 2**).

### 3.1 Pie chart visualization

Pie charts illustrate the relative abundance of individual clonotypes within a sample. Each slice represents each clonotype with its size proportion of its share among total receptor reads. A polyclonal repertoire (healthy or naïve individuals) displays a chart with numerous, small, evenly sized slices, indicating high diversity. In contrast, an oligoclonal repertoire, observed during drug-induced skin rush, infection, vaccination, or tumor response, shows a few dominant slices representing expanded antigen-specific clones. Comparing pie charts from different samples or timepoints provides an immediate visual cue to clonal expansion or immune focusing events. **Figure 2A** exemplifies this comparison: the left pie chart, representing a healthy person, demonstrates a highly diverse and polyclonal repertoire, characterized by a vast number of tiny slices, reflecting a balanced immune system ready to respond to a wide variety of potential antigens. The right pie chart, representing the skin-rash patient, shows a clonally expanded and oligoclonal repertoire (unpublished data), where the chart is dominated by nearly 10 dominant T-cell clones (large slices), indicating a focused, intense immune response, likely driven by the drug, that has led to the massive proliferation of a small set of specific T-cells and a significant loss of overall diversity. This visual difference clearly illustrates how immunopathology can shift the T-cell population from a broad, healthy spectrum to a narrow, disease-associated one.

### 3.2 Diversity indices

While pie charts are qualitative, diversity indices quantify repertoire complexity. The Shannon index integrates richness and evenness, with higher values reflecting a broader and more balanced clonotype distribution. The Simpson index emphasizes dominant clones, decreasing when a few lineages dominate. The Gini coefficient measures inequality: a value near zero indicates uniform clone sizes, whereas a value near one denotes strong clonal dominance. Collectively, these metrics quantify immune balance versus activation-driven skewing as shown in **Figure 2B**, displaying a gradient of T-Cell Receptor (TCR) diversity across samples from 23 colorectal cancer patients, measured using the inverse Simpson’s diversity index, where higher values denote greater diversity (17). Diversity is lowest in the primary tumors, indicating a constrained T-cell population, then significantly increases in metastasis-positive lymph nodes (LN (meta+), suggesting a broader immune response recruitment. The highest diversity is observed in the metastasis-negative lymph nodes (LN (meta-), which is significantly higher than in LN (meta+) (P < 0.0001). This suggests that the presence of metastases correlates with a localized reduction in the breadth and variety of the T-cell repertoire.

### 3.3 Clonotype frequency dynamics

Temporal analysis of clonotype frequencies tracks immune activation and memory formation as well as immune inhibition. By comparing repertoires over time, we can compute fold-changes in clonotype abundance, identifying clones that expand (antigen response) or reduce (inhibition of certain reactions), or persist (memory). **Figure 2C** illustrates this in two cases of Kawasaki disease (KD), showing expanded B-cell clone frequencies in blood from the acute phase (pre-IVIG treatment) to the recovery phase (post-treatment). In the acute phase, multiple distinct clones reach 1.5–2.0% frequency. Following IVIG therapy, these expanded clones nearly disappear, suggesting that IVIG effectively suppresses or eliminates pathogenic B-cell clones, potentially driving the acute disease phase (18).

## 4 Immune-mediated adverse drug reactions

Immune-mediated adverse drug reactions (IM-ADRs) account for a small fraction of all adverse drug events, yet they are among the most serious because they arise from activation of the adaptive immune system rather than predictable drug pharmacology. Unlike dose-dependent toxicities, IM-ADRs are often unpredictable, may occur even with minimal exposure, and can be life-threatening. These reactions are categorized into two groups. Immediate reactions are usually mediated by drug-specific IgE antibodies and occur within minutes to hours after exposure and often manifest acute clinical features such as urticaria, angioedema, bronchospasm, or anaphylaxis in severe cases. Delayed reactions, on the other hand, emerge days to weeks after drug initiation and involve a wide spectrum of immune mechanisms and clinical manifestations. These range from mild exanthematous eruptions to severe, multisystem syndromes such as Stevens–Johnson syndrome (SJS), toxic epidermal necrolysis (TEN) drug reaction with eosinophilia and systemic symptoms (DRESS) as well as drug-induced liver injury (DLI) (19).

### 4.1 IM-ADRs targeting skin

SJS and TEN are severe cutaneous adverse reactions characterized by fever, mucosal erosions, and widespread epidermal detachment with mortality in TEN reaching as high as 50%. The pathogenesis involves cytotoxic CD8^+^ T cells and NK cells releasing granulysin, a key mediator that drives keratinocyte apoptosis (20). Strong genetic predispositions have been identified, most notably the association between carbamazepine-induced SJS/TEN and HLA-B*15:02 in Han Chinese (10). This reaction exemplifies the pharmacological interaction (p-i) concept, in which carbamazepine is suspected to bind noncovalently to the HLA-B*15:02–TCR interface, then triggering activation of autoreactive CD8^+^ T cells without requiring drug metabolism or antigen processing (21). Some examples of drug-HLA association related to IM-ADRs are summarized in **Table 1**.

**Table 1:** Drug-HLA interactions in adverse drug reactions (Skin, DILI, Agranulocytosis).

Drug	HLA allele(s)	Adverse reaction(s)	Notes/evidence	Risk odds ratio
Carbamazepine	HLA-B*15:02 HLA-A*31:01	SJS/TEN DRESS, hypersensitivity	Strong, widely replicated (esp. East Asians); testing recommended. Reported in Europeans and Japanese.	39-2504 32-58
Allopurinol	HLA-B*58:01	SJS/TEN, DRESS	Elevated risk across populations.	118-580
Abacavir	HLA-B*57:01	Hypersensitivity syndrome	Clinically actionable, routine pre-treatment screening.	117-960
Vancomycin	HLA-A*32:01	DRESS	Robust association in European cohorts.	13-24
Dapsone	HLA-B*13:01	Dapsone hypersensitivity/DRESS	Strong predictor in multiple ethic groups.	21
Methazolamide/sulfonamides	HLA-B*59:01	SJS/TEN (ocular/skin)	Reported mainly in East Asian populations.	16-1322
Antithyroid drugs (carbimazole, methimazole, PTU)	HLA-B*38:02 HLA-DRB1*08:03 HLA-B*27:05	Agranulocytosis/severe neutropenia	Consistent associations in East Asian studies.	41-265
Clozapine	HLA-DQB1 variants (rs113332494, nearby)	Neutropenia, agranulocytosis	Reported in European ancestry cohorts.	10-16
Flucloxacillin	HLA-B*57:01	DILI (cholestatic liver injury)	Strongest known HLA-DILI association; risk markedly elevated.	37-86
Amoxicillin-clavulanate	HLA-DRB1*15:01 HLA-DQA1*01:02 HLA-DQB1*06:02	DILI (cholestatic/mixed injury)	One of the most common causes of DIL; HLA class II variants implicated.	23
Lapatinib	HLA-DQA1*02:01	DILI (hepatocellular injury)	Association observed in clinical trials.	9
Lumiracoxib	HLA-DRB1*15:01 HLA-DQA1*01:02	DILI (severe hepatotoxicity)	Reported in European cohorts; drug withdrawn in many regions.	5
Ticlopidine	HLA-A*33:03	DILI (cholestatic type)	Reported in Japanese patients.	13-37

The frequencies of HLA alleles vary substantially across different ethnic groups. Additionally, the presence or absence of specific modifier(s) can affect the odds ratios. Therefore, predicted values should be assessed and clinically validated for each population, country, or region.

Another T cell-mediated syndrome, drug reaction with eosinophilia and systemic symptoms (DRESS), presents with rash, fever, lymphadenopathy, eosinophilia, and multi-organ involvement (22). Unlike SJS/TEN, DRESS typically has a longer latency period, often appearing 2–6 weeks after drug initiation. The liver, kidney, lung, and heart are commonly affected. Although its mortality rate (estimated at 5–10%) is lower than that of TEN, severe organ dysfunction can lead to long-term morbidity. Reactivation of latent viruses, particularly human herpesvirus 6 (HHV-6), is frequently observed and thought to amplify the immune response, further complicating disease progression (23). Genetic risk factors such as HLA-B*58:01 with allopurinol-induced DRESS, underscore the importance of host factors in susceptibility and highlight the potential for prevention through genetic screening.

### 4.2 IM-ADRs targeting liver and other tissues

In addition to cutaneous syndromes, immune-mediated adverse drug reactions can also affect vital organs such as drug-induced liver injury (DILI) being a prominent example. In Europe, two antibiotics, flucloxacillin and amoxicillin–clavulanate, account for a substantial proportion of DILI cases. Flucloxacillin-induced DILI is strongly associated with HLA-B*57:01, conferring estimated ~80-fold increased risk to carriers, whereas the pathogenic mechanisms of amoxicillin–clavulanate–induced DILI is still unclear, but likely to be linked to multiple HLA class I and II alleles (24, 25). Covalent drug–protein adducts have been detected in human liver tissue, while *in vitro* studies demonstrate that flucloxacillin can alter the peptide landscape presented by HLA-B*57:01, thereby activating cytotoxic T cells. Other organ-specific IM-ADRs include antithyroid drug–induced agranulocytosis, a severe hematologic reaction strongly associated with HLA-B*38:02 and HLA-DRB1*08:03 (26). Clinically, this condition is characterized by profound neutropenia that predisposes patients to severe infections, often requiring hospitalization and immediate drug withdrawal. Together, these examples demonstrate that IM-ADRs are not limited to the skin but can also target critical organ systems, where they may provoke fatal immune responses. 3.

### 4.3 Clinical translation

Routine HLA-B*57:01 screening before abacavir treatment, HLA-B*15:02 testing prior to carbamazepine uses in Southeast Asia, and HLA-B*58:01 testing before initiating allopurinol is now recommended or mandated in many countries. These interventions demonstrate how integrating immunopharmacogenomics into clinical workflows can prevent life-threatening IM-ADRs, improve patient safety, and reduce healthcare costs (27). However, the abacavir example also underscore why developing reliable predictive biomarkers for IM-ADRs is inherently challenging. A major factor behind the success of HLA-B*57:01 screening is the exceptionally high predictive performance: abacavir hypersensitivity syndrome has a 100% negative predictive value (NPV), meaning the absence of the allele effectively rules out the reaction, and a relatively high positive predictive value (PPV) of approximately 55% (28). Other IM-ADRs show some practical limitations of HLA-based screening. For example, in flucloxacillin-induced liver injury, nearly 14,000 patients would need to be screened to prevent a single case (29), rendering the wide-spread implementation impractical. Hence, while HLA-B*57:01 testing for abacavir remains a landmark achievement in precision medicine, it represents the exception rather than the general rule. For most drugs, HLA associations alone are insufficient, and clinically useful prediction will require integrating additional biomarkers, such as functional immune assays, or multi-omics risk models. These multi-dimensional approaches hold promises for extending the success of immunopharmacogenomics beyond the rare cases exemplified by abacavir.

## 5 Cancer treatment

Cancer immunotherapy, particularly a class of drugs known as immune checkpoint inhibitors (ICIs), has revolutionized the treatment of many cancer types. Unlike traditional cytotoxic or molecular-targeted therapies that act directly on tumor cells, ICIs harness the patient’s immune system by blocking immune-inhibitory pathways (molecules and cells) that restrain T-cell activation (11). Two key checkpoint pathways involve cytotoxic T-lymphocyte antigen-4 (CTLA-4) and the programmed death-1 (PD-1)/programmed death-ligand 1 (PD-L1) axis. CTLA-4, expressed on activated conventional T cells and constitutively on regulatory T cells (Tregs), acts as a negative regulator of T-cell activation by competing with the costimulatory receptor CD28 for binding to B7 ligands (CD80/CD86) on antigen-presenting cells (APCs). This competition attenuates the costimulatory signals required for full T-cell activation, thereby dampening early immune responses in lymphoid tissues (30). PD-1, upregulated on activated T cells, interacts with its ligands PD-L1 or PD-L2, which are expressed on tumor cells, stromal cells, and antigen-presenting cells. This engagement transmits inhibitory signals that suppress T-cell proliferation, cytokine production, and cytotoxic activity within the tumor microenvironment. Monoclonal antibodies targeting these checkpoint molecules can restore T-cell activity and produce durable clinical responses, even in patients with advanced cancers (31). However, unleashing T-cell activity can also provoke immune-related adverse events (irAEs), in which the immune system attacks normal tissues. irAEs can affect any organ, with common manifestations including colitis, dermatitis, hepatitis, endocrinopathies, and pneumonitis (12, 32). While most irAEs are manageable with immunosuppressive therapy, severe events can be life-threatening and sometimes lead to treatment discontinuation. Immunogenomic approaches, such as HLA typing, TCR repertoire analysis, and multi-omics profiling, are increasingly being explored to identify patients at higher risk of irAEs and to predict which individuals are most likely to benefit from ICIs (33, 34). These strategies aim to optimize the balance between therapeutic efficacy and safety, advancing personalized cancer immunotherapy.

We reported an 80-year-old melanoma patient who developed myocarditis, polymyositis, and myasthenic crisis after a single infusion of nivolumab (anti-PD-1 antibody). The patient had pre-existing anti-acetylcholine receptor antibodies, which rose sharply following treatment. TCR sequencing revealed multiple T cell clonotypes infiltrating to muscle tissue, suggesting the unleashing of a possibly pathogenic immune response (35). This case highlights a severe and potentially life-threatening complication, showing that ICIs can precipitate latent autoimmune diseases into acute crises. These examples underscore the urgent need for predictive biomarkers of immune-related adverse event risk. Immunopharmacogenomics offers a promising framework to address the challenges. By integrating HLA typing, baseline autoantibody screening, and TCR/BCR repertoire sequencing, it may be possible to identify patients harboring latent autoreactive clones prior to therapy. Such strategies could allow patients to stratify by risk, tailor treatment regimens, and implement closer monitoring, providing critical insights into how immune activity can be safely and effectively managed.

**Figure 3:**
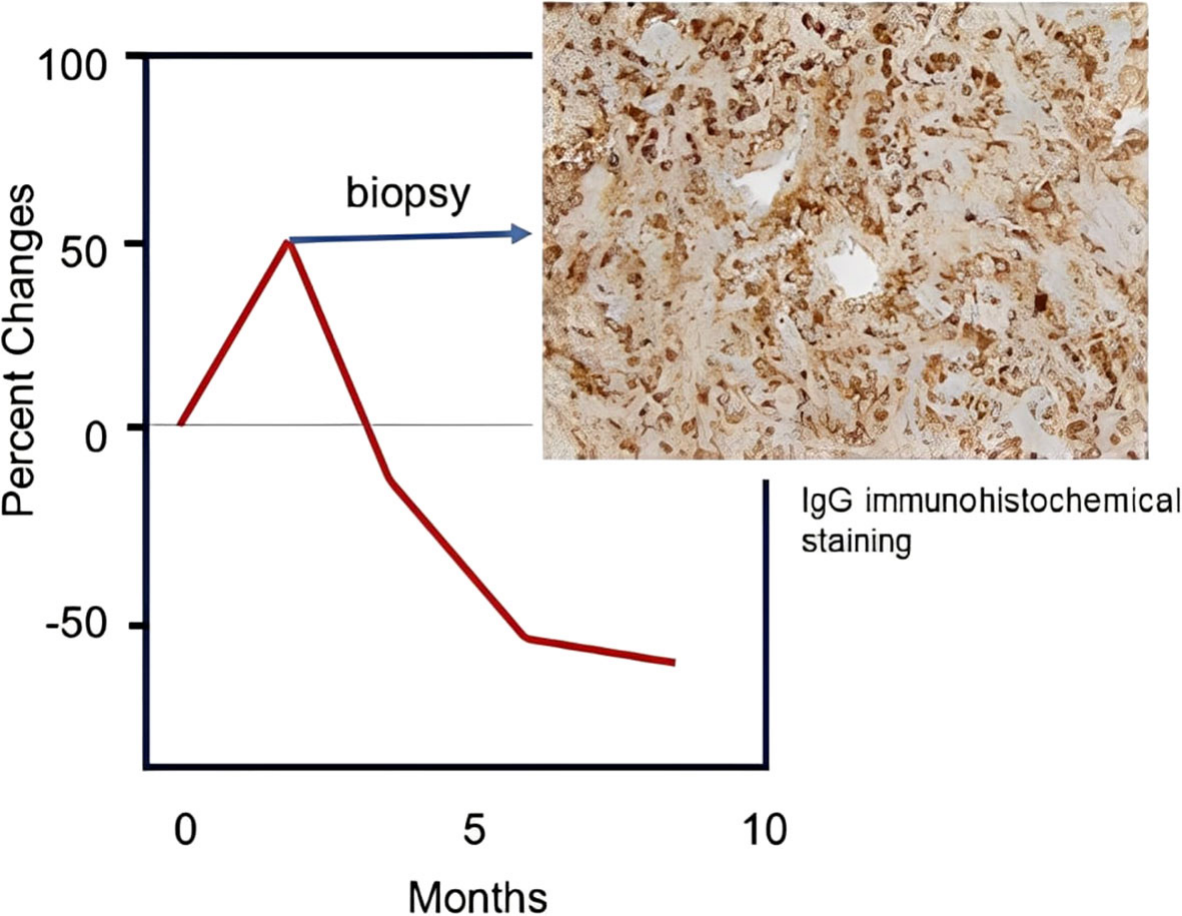
Histopathological analysis of a tumor with pseudoprogression. Red line indicates changes in tumor size, showing approximately 50% increase two months after the initiation of dual immune checkpoint inhibitor (ICI) therapy with durvalumab (anti–PD-L1 antibody) and tremelimumab (anti–CTLA-4 antibody) in a case of metastatic breast cancer. Immunohistochemical analysis of the tumor at two months demonstrated strong IgG staining within the tumor region, consistent with the presence of plasma cells (CD138-positive). (Adapted from Santa-Maria et al. (36)).

A pilot study of dual ICI therapy with durvalumab (anti–PD-L1 antibody) and tremelimumab (anti–CTLA-4 antibody) in metastatic breast cancer provides a good example of how immunopharmacogenomic profiling can inform treatment evaluation (36). In this trial, objective responses were observed in 43% of triple-negative breast cancer (TNBC) cases. Among responders, a small subset of T cells revealed significant clonal expansion within the tumor following immunotherapy, indicating robust stimulation of anti-tumor immune response (36). Notably, as shown in **Figure 3**, histopathological analysis of a tumor exhibiting pseudoprogression after two months of therapy identified dense plasma cell infiltration accompanied by intense IgG deposition in the tumor microenvironment. B-cell receptor (BCR) repertoire analysis further demonstrated the emergence of novel clonotypes, reinforcing the interpretation that the apparent tumor enlargement was driven by immune infiltration rather than true tumor progression. Taken together, these findings highlight the value of immunopharmacogenomic approaches in refining response assessment, clarifying mechanisms of sensitivity and resistance, and distinguishing immune-related radiographic changes from pseudo-tumor progression.

The association between clonal T-cell dynamics and therapeutic outcome has also been demonstrated in lung cancer. In a longitudinal study of 27 patients treated with anti–PD-1 monotherapy or combined checkpoint blockade, durable responders consistently revealed sustained oligoclonal expansion of dominant CD8^+^ T-cell clones in peripheral blood and, in one cancer tissue examined (37). For example, in a patient with squamous cell lung cancer who achieved a pathologic complete response, a single clonotype accounted for nearly 20% of all TCR reads in the metastatic lesion by day seventeen of therapy. The same clonotype, initially present at 6.1% in peripheral blood prior to treatment, expanded to 24.3% by week forty-eight. The persistence of one or two dominant T cell clones at high frequency in peripheral blood appears to be a hallmark of durable clinical benefit. Even in patients lacking baseline samples, long-term responders consistently displayed sustained oligoclonal expansions (37), like prior findings from adoptive T cell therapy where durable benefit was linked to persistence of infused clones. In contrast, non-responders showed no evidence of persistent clonal expansion. These findings suggest that long-term clinical benefit from checkpoint blockade requires not only the initial recruitment of tumor-reactive T-cell clones but also their durable maintenance. Accordingly, longitudinal TCR repertoire sequencing analysis may serve as a practical biomarker to distinguish durable responders early during the course of therapy.

Further evidence comes from colorectal cancer. In our study, TCR repertoires were compared between primary tumors and tumor-draining lymph nodes (TDLNs). Metastasis-positive TDLNs showed reduced diversity and greater clonal overlap with primary tumors compared to metastasis-negative nodes. Principal component analysis further revealed distinct clustering of metastasis-positive TDLNs, suggesting enrichment of tumor-reactive clones (17). Similar patterns were reported in melanoma. For example, response to PD-1 blockade was linked to intratumoral oligoclonal T cell expansion accompanied by increased expression of cytolytic effector genes, including granzyme A and perforin (38). Taken together, these studies point to a possible unifying principle: effective ICI therapy is marked by oligoclonal focusing of the TCR repertoire and enhanced cytotoxic activity—hallmarks that immunopharmacogenomics can detect with high sensitivity.

## 6 Graft versus host disease

Graft-versus-host disease (GVHD) remains a major complication of allogeneic hematopoietic stem cell transplantation (HSCT). It occurs when donor T cells recognize host alloantigens and induce an immune response that damages host tissues, most frequently the skin, gastrointestinal tract, liver, and lung. Acute GVHD typically develops within the first few months after transplantation, whereas chronic GVHD develops later and often compromises long-term quality of life (39). Notably, the therapeutic benefits of HSCT, namely the graft-versus-leukemia (GVL) effect, are inseparable from the same alloreactivity that drives GVHD, rendering the condition an enduring immunological paradox (40). Prior to engraftment, HSCT recipients undergo myeloablative or reduced-intensity conditioning with chemotherapy and/or radiation. While these regimens eradicate malignant cells and suppress host immunity, they also cause widespread tissue injury, resulting in activation of inflammatory signals (41). Immunopharmacogenomic profiling of the T-cell repertoire has provided important insights into the mechanisms driving GVHD. Using next-generation sequencing of TCRs, we mapped the clonal architecture of TCRα and TCRβ repertoires following HSCT (42). These analyses consistently demonstrated that HSCT procedure markedly reduce repertoire diversity. Both TCRα and TCRβ chains exhibit diminished variability compared with pre-transplant baselines, reflecting profound lymphodepletion and the constraints of early immune reconstitution. Importantly, it is not simply the number of T cells that predicts GVHD, but the configuration of the repertoire. Broadly diverse TCR repertoire is associated with balanced immune reconstitution and reduced GVHD risk, whereas a clonally- or oligoclonally-expanded repertoire, dominated by a limited number of expanding clones, is linked to pathogenic alloreactivity and more severe GVHD (43).

**Figure 4:**
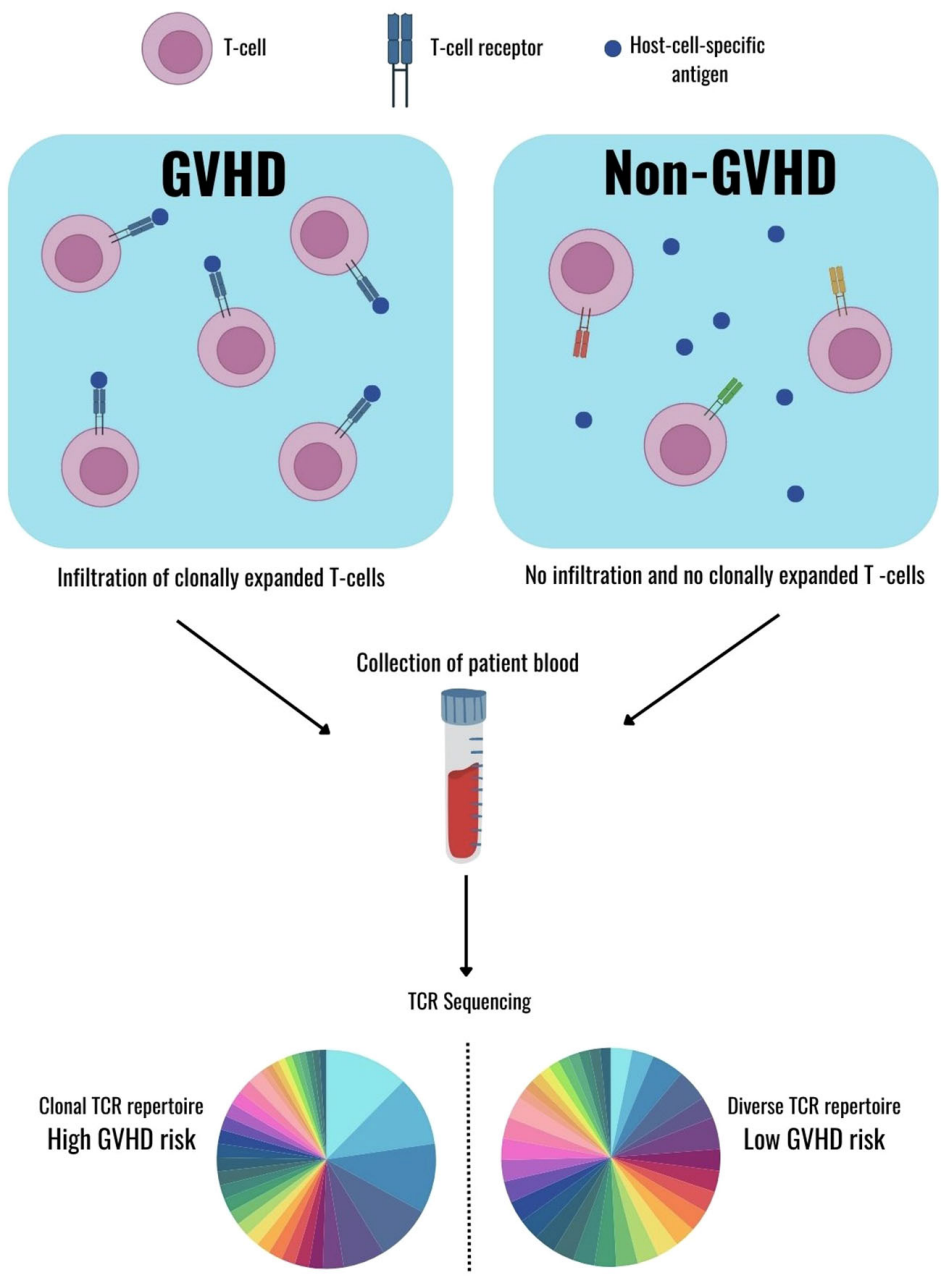
Immune characterization of GVHD. Donor T cells recognize host-specific antigens and undergo clonal expansion within affected tissues. These expanded clones can be detected in peripheral blood after HSCT or BMT, even before the onset of acute GVHD symptoms. Broad T-cell repertoire diversity is generally protective for GVHD, whereas oligoclonal dominance promotes pathogenic alloreactivity and increases GVHD risk (adapted from 3).

As illustrated in **Figure 4**, GVHD develops when donor-derived T cells recognize host-specific antigens within target tissues (3). Antigen recognition drives T-cell activation and clonal proliferation at these sites, which can be detected as oligoclonal expansions in peripheral blood following hematopoietic stem cell or bone marrow transplantation. Notably, such repertoire shifts often precede the onset of clinical symptoms, highlighting the potential of TCR sequencing as an early, non-invasive biomarker for GVHD monitoring. Patients with acute GVHD exhibit significantly higher cumulative frequencies of the top ten most abundant TCRB clonotypes compared with non-GVHD patients (42). The association between clonal expansion and GVHD is more apparant in patients without relapse. Among non-relapsed recipients, both TCRA and TCRB top-clone proportions are significantly elevated in GVHD compared with non-GVHD patients, whereas no such difference is observed in relapsed individuals, likely because leukemia recurrence dilutes or obscures repertoire-based signatures (42). Larger cohort studies have confirmed these observations, showing that early post-transplant clonal bursts, reflected in enrichment of dominant clones, are probably predictive of acute GVHD, even when early-onset cases are excluded (44). Thus, clonal expansions represent not merely downstream consequences but early hallmarks of pathological alloreactivity.

Murine models provide deeper mechanistic insight into GVHD pathogenesis. Dominant clonotypes detected in peripheral blood are often shared with target organs such as the gut, liver, and lungs as early as day seven after transplantation, indicating that blood sampling can serve as a dependable proxy for tissue infiltration (45). Interestingly, most of these dominant clones are absent from the pre-transplant donor repertoire, implying their emergence from rare or nascent rearrangements that undergo selective expansion under allogeneic pressure. Repertoire dynamics are influenced by external factors. Cytomegalovirus (CMV) reactivation, a common complication following HSCT, can profoundly reshape immune recovery. Patients experiencing CMV reactivation exhibit skewed TCR repertoires toward oligoclonality, expansion of effector-memory CD8^+^ T cells, and an increased risk of GVHD (46). In contrast, early high donor T-cell chimerism after cord-blood transplantation is associated with greater repertoire diversity and a lower incidence of GVHD (47). It is indicated that GVHD risk is not solely determined by alloantigen recognition, but it is also shaped by other factors such as infection history, donor source, and relapse status, which collectively influence immune reconstitution.

These findings highlight an important translational opportunity. By incorporating frequent and longitudinal TCR repertoire monitoring and diversity metrics, clinicians may be able to predict GVHD risk before clinical symptoms appear and then fine-tune immunosuppressive regimens more precisely. Because peripheral blood TCR sequencing can detect oligoclonal expansions and contractions in repertoire diversity days or weeks prior to symptomatic GVHD, it provides a critical window for preemptive intervention.

## 7 Autoimmune diseases

Autoimmune diseases arise from dysregulated adaptive immune responses against self-antigens. Extensive repertoire analyses and single-cell immunogenomic profiling have revealed that disease progression is frequently marked by clonal expansions of autoreactive T and B cells, which are often influenced by specific HLA genotypes within affected tissues (48). Across diverse autoimmune pathologies, a defining feature is thus the antigen-specific clonal expansion of lymphocytes, rather than generalized inflammation. Immunopharmacogenomics provides tools to capture these repertoire shifts with high resolution, linking clonotype patterns to underlying disease mechanisms and evolution, and potential therapeutic targets.

The immune system employs multiple layers of tolerance to prevent autoreactivity. Central tolerance, occurring in the thymus and bone marrow, eliminates lymphocytes with high affinity for self-antigens through negative selection. Peripheral tolerance, mediated by regulatory T cells (Tregs) and immune-privileged sites, constrains autoreactive clones that escape central deletion (49). Failures in these tolerance mechanisms, shaped by genetic susceptibilities (e.g., HLA alleles), environmental triggers (e.g., infections, microbiome alterations, chemicals), and dysregulated immune checkpoints, permit the expansion of autoreactive clones. Importantly, immunopharmacogenomic studies demonstrate that these expansions are nonrandom, often exhibiting convergent V–J gene usage and recurring CDR3 motifs, strongly supporting antigen-driven selection as a central driver of autoimmunity.

### 7.1 Crohn’s disease

Crohn’s disease (CD) provides a compelling example of how repertoire sequencing can uncover disease-driving clonotypes. Deep TCR sequencing of mucosal biopsies from ulcerative tissues from patients with recurrent CD demonstrated that TCR diversity was significantly lower than in matched peripheral blood mononuclear cells, reflecting localized clonal T-cell expansions (50). Notably, the CASSWTNGEQYF CDR3 clonotype with TRBV10-1/TRBJ2–7 combination was markedly enriched, representing 7.0–28.9% of the TCR repertoire sequences in four clinically severe cases (higher Rutgeerts scores) among twelve patients examined (50). Collectively, these clinical and molecular findings illustrate that repertoire sequencing can identify T-cell clonotypes that may be associated with disease severity and provide information relevant to therapeutic target.

Subsequent studies reinforced these observations, showing reduced TCR diversity and focal clonal expansions in inflamed intestinal mucosa of CD patients relative to healthy controls (51). Furthermore, the persistence of expanded clonotypes following surgery predicted disease relapses, highlighting their potential as non-invasive biomarkers for recurrence risk stratification (52). Together, these studies indicate that Crohn’s disease is not merely a diffuse inflammatory disorder but is instead characterized by selective expansions of mucosa-resident autoantigen-specific T-cells.

### 7.2 Ankylosing spondylitis

HLA-B27 positivity is observed in 90-95% of patients with ankylosing spondylitis (AS), psoriatic arthritis and acute anterior uveitis, of European descent, compared with only 6–8% in the general population. The presence of HLA-B27 increases the risk of developing AS by 20–50 times, although the HLA allele is not sufficient on its own, as only a fraction of carriers develops the disease. Recent repertoire studies consistently identified enrichment of TRBV9+ CD8+ T cells in patients with AS (53). These TRBV9+ cells often displayed an effector-memory phenotype and produced pro-inflammatory cytokines. Building on this discovery, researchers developed a monoclonal antibody targeting TRBV9 to selectively deplete T cells expressing this Vβ segment. In one case, a man with severe, treatment-refractory AS, who had failed anti-TNF therapy, received this TRBV9-specific antibody. Clinical improvement became evident within weeks, including marked reductions in inflammatory back pain, enhanced spinal mobility, and normalization of C-reactive protein levels. Remarkably, only three doses per year were required to maintain remission, and the patient remained relapse-free for four years. Immunosequencing confirmed selective depletion of TRBV9-positive T cells without inducing global immunosuppression (53). This case illustrates a paradigm shift in therapy which means rather than broadly suppressing the immune system, treatments can now selectively target T-cell clonotypes including disease-driving T cells through repertoire profiling. It exemplifies the promise of personalized medicine, in which immunopharmacogenomics guides patient- or disease- specific therapy.

### 7.3 Systemic lupus erythematosus and lupus nephritis

Systemic lupus erythematosus (SLE) is a multifactorial autoimmune disease influenced by both genetic and environmental factors. Certain HLA class II alleles, particularly HLA-DR2 (DRB1*15) and HLA-DR3 (DRB1*03), are indicated the association with increased susceptibility to SLE across multiple populations, while other HLA alleles can modulate disease manifestations, autoantibody profiles, and severity, pointing out the central role of CD4^+^ T helper cells in disease pathogenesis (54, 55).

Sequencing of peripheral blood from patients with lupus nephritis (LN) revealed significantly reduced TCR diversity compared with healthy controls, characterized by expansion of dominant clonotypes (56). Distinct biases in TRBV/TRBJ combinations, for example, enrichment of TRBV12-5/TRBJ2–1 and depletion of TRBV10-3/TRBJ2-6, distinguished SLE patients from controls, suggesting that specific autoreactive TCR clonotypes contribute to tissue pathology. These repertoire signatures highlight the potential for non-invasive biomarkers for LN, addressing a critical need given the invasiveness of renal biopsy and the scarcity of reliable prognostic tools. Complementary evidence came from a single-cell transcriptome analysis of PBMC alongside paired TCR and BCR repertoires in SLE. Compared with healthy controls, SLE patients showed accumulation of neutrophils, macrophages, and dendritic cells, with differentially expressed genes enriched in pro-inflammatory NF-KB and TNF signaling pathways. These innate immune alterations were concordant with expansion of both TCR and BCR clonotypes and biased V(D)J usage across TCR α/β and BCR IGH/IGK/IGL chains (57).

Further granularity comes from studies of neuropsychiatric lupus (NPSLE), which affects up to 40% of SLE patients. Using the lupus-prone MRL/lpr mouse model, researchers found that TCR repertoires in the choroid plexus were oligoclonal and distinct from those in the spleen and salivary glands, suggesting selective recruitment of autoreactive T cells into the central nervous system (CNS) (58). Importantly, these clonal expansions observed in the brain were not random spillovers from systemic lymphoproliferation but reflected tissue-specific T-cell clonal recruitment and expansion, indicating the potential value of immunogenomic profiling in elucidating organ-specific manifestations of lupus.

### 7.4 Type 1 diabetes

The HLA region accounts for approximately 50% of the genetic risk for Type 1 diabetes (T1D) and HLA class II molecules, especially HLA-DR and HLA-DQ, are indicated to be the most significant contributors to susceptibility (59). Among various class II haplotypes, DRB1*03:01-DQA1*05:01-DQB1*02:01 and DRB1*04:01/04:02-DQA103:01–DQB1*03:02, are known to be high-risk alleles (60). A large-scale analysis of >2×10^8^ TCRβ sequences across naive, memory, regulatory, and stem-cell–like CD4^+^ T-cell subsets revealed that T1D patients exhibit abnormally shortened CDR3 loops across all subsets (61). This shortening was attributed to defects in VDJ recombination, including excessive nucleotide deletions and reduced insertions during rearrangement, rather than altered thymic selection. The phenomenon was observed even in unproductive TCR sequences, indicating that it originates at the recombination stage, independent of tolerance checkpoints.

Functionally, autoreactive TCRs isolated from insulin- and GAD65-specific CD4^+^ T cells in T1D patients had shorter CDR3s than antiviral TCRs or TCRs from healthy controls, suggesting that shortened CDR3s increase the probability of recognizing self-peptide/HLA complexes (61). Beyond abnormal shortening, TCR repertoires in T1D patients also exhibited greater clonotype sharing compared with healthy donors, reflecting convergent selection of disease-driving clonotypes across individuals. Hence, these findings support a model in which aberrant rearrangement skews the TCR repertoire toward autoreactivity, predisposing patients to β-cell autoimmunity. TCR sequencing also highlighted a biomarker strategy in T1D. Current clinical prediction primarily relies on autoantibody screening, against insulin, GAD65, IA-2, and ZnT8, but these antibodies are indirect indicators of T-cell activity. Because TCR sequences uniquely define antigen specificity, high-throughput TCR profiling offers a more direct method for assessing autoreactive T cells in both blood and pancreatic tissue (62).

## 8 Food allergies

Food allergies affect 6–8% of children and about 5% of adults worldwide, with peanut, milk, and egg being the most clinically significant allergens. Allergen-specific IgE bound to FcϵRI on mast cells and basophils mediates immediate hypersensitivity reactions, which can lead to life-threatening anaphylaxis (63). Immunotherapy strategies such as oral immunotherapy (OIT) have emerged as promising interventions, aiming to induce desensitization or sustained unresponsiveness (64). Immunopharmacogenomic profiling may offer the potential to personalize these therapies by identifying genetic and immune determinants of treatment response.

### 8.1 TCR clonality in peanut allergy

One of the earliest demonstrations of clonotypic T-cell expansion in peanut allergy came from studies of HLA class II–matched siblings. In children with peanut allergy, *in vitro* stimulation with peanut extract induced oligoclonal or clonal skewing of T cells with TCR-Vβ11 sequence, whereas non-allergic siblings showed no clonal expansion of specific T cells (65). This selective expansion suggested that allergen-driven clonal T-cell responses contribute to the pathogenesis of peanut allergy. These findings laid the groundwork for repertoire sequencing approaches to identify allergen-reactive clonotypes with potential diagnostic and prognostic value.

### 8.2 T cell subsets in food immunotherapy

Subsequent studies have broadened the view beyond a simple Th2/Th1 dichotomy. High-dimensional profiling in mechanistic investigations of OIT has indicated critical roles for T follicular helper cells (Tfh, particularly Tfh2), regulatory T cells (Tregs), tissue-resident memory T cells (TRM), γδ T cells, and invariant NKT cells (66). Immunotherapy elicits a spectrum of cellular changes, including attenuation or functional silencing of allergen-specific Th2 cells, expansion of Tregs, and modulation of Tfh subsets that support B cells help and class switching (66). These findings demonstrate that tolerance induction is not a simple conversion of Th2 into Th1, but rather a coordinated remodeling of the allergen-reactive T-cell ecosystem.

### 8.3 Epitope-defined and single-cell repertoire profiling

A central challenge in applying immunopharmacogenomics to food allergy is the extreme rarity of allergen-specific T cells in peripheral blood as well as the biochemical complexity of crude allergen extracts (67). To date, only ~21 food allergens have well-defined T-cell epitopes, far fewer than in infectious disease contexts (68). Recent advances in epitope mapping and single-cell technologies are helping to close this gap. Activation-induced marker assays (AIM/ARTE), HLA class II tetramers, and higher-order multimers (e.g., dodecamers), together with single-cell RNA sequencing, enable highly specific capture of rare allergen-reactive CD4^+^ T cells and TCR clonotypes (69, 70). These tools reveal that, in OIT, baseline characteristics of allergen-specific T cells, including their phenotypic programs and clonal architecture, can stratify patients with a likelihood of response. Moreover, successful OIT is frequently accompanied by contraction or functional silencing of Th2-skewed clones and a shift toward anergic or regulatory phenotypes (68). Collectively, epitope-defined, repertoire-guided single-cell approaches are emerging as essential next-generation platforms for precision medicine in food allergy, enabling patient stratification, response prediction, and pharmacodynamic monitoring of OIT outcomes.

### 8.4 Antibody isotypes and BCR repertoires

While T cells orchestrate the allergic cascade, B-cell repertoires and antibody isotypes provide essential humoral readouts. Successful immunotherapy is consistently associated with a decline in allergen-specific IgE and a rise in IgG4 and IgA, antibody classes that block allergen binding (71). These serological signatures, long recognized in clinical studies, can now be analyzed in detail at the genetic level. In our study, BCR sequencing was applied to children with peanut allergy who received OIT. OIT decreased IgE expression and repertoire diversity, consistent with reduced IgE-producing cells and oligoclonal expansion of protective B-cell clones (72).

## 9 Organ transplant rejection

Organ transplantation remains the definitive therapy for end-stage organ failure, yet long-term graft survival continues to be limited by immune-mediated rejection. Despite advances in HLA matching and immunosuppressive therapy, up to 50% of kidney allografts fail within 10 years, underscoring the need for more effective tools to predict, diagnose, and monitor rejection (73). **Table 2** summarizes key repertoire features that help distinguish stable from rejecting kidney transplant recipients. Unlike GVHD, organ rejection reflects the reverse immunological direction. Recipient lymphocytes mount an immune response against the donor organ (74). Both T cell–mediated rejection (TCMR) and antibody-mediated rejection (ABMR) remain major contributors to chronic graft loss, even under potent immunosuppressive regimens (75). High-throughput sequencing of TCR and BCR repertoires has revealed that rejection episodes are characterized by oligoclonal expansions of donor-reactive clones, detectable both in graft tissue and in peripheral blood. On the B-cell side, repertoire profiling has demonstrated uneven IGHV gene usage, such as enrichment of IGHV3–23 in rejecting patients, providing molecular insights that complement conventional donor-specific antibody (DSA) testing (73).

**Table 2:** Key immune repertoire features distinguishing stable and rejecting kidney transplant recipients.

Feature	Stable recipients	Rejecting recipients	Reference
BCR diversity (pre-transplant)	Lower baseline diversity	Higher baseline diversity predicts later rejection.	(73)
BCR repertoire (post-transplant)	Diversity increases over time	Progressive loss of diversity with oligoclonal expansion of dominant clones.	(73)
IGHV usage	Heterogeneous	Enrichment of IGHV3–23 and other biased segments, consistent with convergent selection.	(73)
TCR fraction (baseline)	Normal/maintained	Lower at baseline, rise sharply at rejection.	(76)
TCR turnover (Morisita index)	Low, stable over time	High turnover during rejection, especially late or ABMR cases.	(76)
TCR clonality	Balanced repertoire	Broad repertoire remodeling; not necessarily dominated by single clone.	(76)
Infection cofounders	Minimal effect	CMV reactivation increases TCR turnover, amplifying rejection risk.	(76)
Clinical integration	Stable repertoire patterns align with biopsy findings.	Clonal expansions align with TCMR/ABMR; sequencing can reclassify ambiguous cases.	(77)
Therapy response	Diversity maintained or recovers with therapy.	Persistence of dominant clones signals refractory rejection.	(77)

### 9.1 TCR repertoire remodeling in rejection

High-throughput sequencing has revealed that rejection is characterized by broad remodeling of recipient T-cell repertoires rather than dominance of single dominant clone. In a large cohort study of 200 kidney transplant recipients, patients who later developed rejection had lower T-cell fractions before transplantation but displayed sharp repertoire turnover at the time of rejection, particularly in late-onset and ABMR cases (76). This turnover was not explained by increased clonality but by wholesale reshaping of the T-cell compartment, suggesting that monitoring repertoire dynamics may enable earlier detection of alloimmune activation earlier than biopsy.

### 9.2 BCR repertoire and antibody-mediated rejection

A key distinction between solid organ rejection from GVHD is the significant role of B cells and antibodies. It is demonstrated that kidney recipients who later experienced rejection exhibited greater pre-transplant B-cell diversity, implying a predisposition toward alloimmunity. During follow-up, patients with rejection showed progressive loss of diversity and oligoclonal expansions, while stable patients revealed maintenance of repertoire diversity. Certain IGHV families, such as IGHV3-23, were consistently enriched in rejecting patients, suggesting convergent antigen-driven selection (73). These findings link repertoire architecture to the generation of donor-specific antibody (DSAs) and establish BCR sequencing as a predictive and mechanistic biomarker search for ABMR.

### 9.3 Clinical application of integrated repertoire profiling

The value of repertoire sequencing extends beyond mechanistic insights. In a prospective study of kidney recipients with ambiguous graft dysfunction, researchers combined TCR and BCR sequencing to complement biopsy. Distinct clonal expansions (e.g., TRG/TRD in TCMR, IGL in ABMR) aligned with histopathology and in some cases reclassified borderline or indeterminate diagnoses (77). Longitudinal analysis further showed that diversity recovery of repertoire after antithymocyte globulin (ATG) therapy paralleled with clinical improvement, whereas persistence of dominant clones signaled refractory rejection. These findings suggest that serial, non-invasive repertoire sequencing could soon supplement or even replace protocol biopsies for rejection monitoring.

### 9.4 Viral triggers and confounders

Repertoire studies also highlight how external factors influence the risk of rejection. For example, CMV reactivation accelerates T-cell turnover and expansion of effector-memory CD8^+^ T cells (76), demonstrating how heterologous viral immunity can exacerbate allo-immune responses. Incorporating such clinical context into repertoire analyses will be essential for accurate interpretation. Taken together, these findings emphasize that solid organ rejection is not simply the inverse of GVHD. While GVHD reflects donor T-cell infiltration into host tissues, organ rejection reflects remodeling of the recipient TCR repertoire alongside BCR-driven antibody responses. The translational priorities differ as well. In GVHD, the goal is to preserve graft-versus-leukemia effects while limiting tissue damage, whereas in organ transplantation the aim is precision immunosuppression, balancing rejection prevention against the risk of infection and malignancy. Integration of immune repertoire sequencing together with analyses of donor-specific antibodies, donor-derived cell-free DNA, and other molecular diagnostics offers a framework for individualized monitoring and therapy, with the potential to extend graft survival and improve patient outcomes.

## 10 Vaccine and drug development

Vaccine performance, durability, and safety are determined by how antigens are processed and presented by HLA molecules and by the extent to which B- and T-cell clones are recruited, expanded, and matured. Modern systems vaccinology integrates early innate and transcriptomic signatures with TCR and BCR repertoire analyses and serology to guide antigen and adjuvant selection, optimize dosing schedule, predict responsiveness, and identify genetic subgroups at risk of non-response or adverse events. The field has moved beyond static antibody titers toward multimodal, longitudinal immune monitoring that can directly inform trial design (78).

### 10.1 Systems vaccinology

Comprehensive profiling of the BNT162b2 mRNA vaccine for COVID19 (SARS-CoV-2 spike (S) protein) in healthy adults revealed that a coordinated immune cascade, comprising early interferon and inflammatory modules, Tfh-mediated B-cell activation, germinal center activity, and class-switched memory formation, predicts both the quantity of neutralizing antibodies and the quality of CD8^+^/Th1 responses (78). These insights enable developers to evaluate adjuvants, dosing regimens, and vaccine platforms using objective early endpoints, rather than waiting for peak antibody titers or clinical outcomes. Transcriptomic and immune signatures measured within the first 1–2 days after each dose, as well as around day 22 post-boost, can suggest optimal dosing intervals and identify potential trade-offs between reactogenicity and immunogenicity.

### 10.2 HLA genetics in efficacy and safety

Persistent non-response to the hepatitis B vaccine showed reproducible associations with HLA-DP. A meta-analysis of HLA-DPB1 alleles identified consistent correlations with antibody responsiveness and non-responsiveness, implicating both peptide presentation and locus-specific expression (79). In practice, epitope panels and adjuvant strategies should be evaluated in silico across diverse global HLA backgrounds, rather than optimized for a single ancestry. Single-cell multi-omics profiling following SARS-CoV-2 mRNA vaccination revealed robust spike-specific CD4^+^ T-cell responses, circulating Tfh cells, and expansion of BCR clonotypes. CD8^+^ T-cell expansion was observed but showed greater inter-individual variability, likely reflecting population-level HLA genetic variations (80). Immune repertoire tracking provides resolution beyond antibody titers: it can differentiate vaccine-induced memory from natural infection, quantify clonal focusing and shared motifs, and enable mechanistic comparisons between homologous versus heterologous booster strategies.

In addition, the narcolepsy signal observed following the AS03-adjuvanted A/H1N1 (Pandemrix) campaign in Europe was concentrated in carriers of HLA-DQB1*06:02. Subsequent genetic studies confirmed this strong association and implicated additional immune and neuronal survival loci (81). This example highlight why pharmacogenovigilance has become a critical component of adjuvanted vaccine platforms.

### 10.3 Personalized cancer vaccines

Patient-specific neoantigen vaccines exemplify the HLA/cancer-specific epitope/TCR paradigm. In the phaseIIb KEYNOTE-942 trial (resected high-risk melanoma), adding the individualized mRNA-4157 (V940) vaccine in addition to pembrolizumab significantly improved recurrence-free survival (RFS) and distant metastasis-free survival (DMFS) compared with pembrolizumab alone (82). Translational studies using TCR sequencing confirmed expansion of vaccine-enriched clonotypes and their infiltration into tumors, linking computational epitope prioritization to functional T-cell responses.

Beyond melanoma, immunopharmacogenomic approaches have been applied to immunologically “cold” tumors, such as pancreatic cancer. In a retrospective study of 16 patients, neoantigen peptide–pulsed dendritic cell (Neo-P DC) vaccines were administered to post-surgery cases including post-recurrence cases. Neoantigen-specific T cells were induced in 81% of patients. In the adjuvant setting, only one recurrence occurred among seven patients during a median follow-up of 61 months, with all patients alive at last observation (83). Among recurrent cases, T-cell responders to neoantigens demonstrated longer survival than non-immune-responders. Notably, combining long HLA class II–binding peptides with short peptides enhanced CD4^+^ T-cell responses and clonal expansions associated with tumor shrinkage (83). This study underscores the power of immunopharmacogenomics to leverage HLA-restricted neoantigen design and monitor TCR repertoire dynamics for therapeutic benefit.

In pancreatic ductal adenocarcinoma (PDAC), a phase I trial tested autogene cevumeran—an individualized uridine mRNA neoantigen vaccine—given sequentially after surgery with atezolizumab and then mFOLFIRINOX (84). The regimen was feasible and well tolerated; among 16 vaccinated patients, 8/16 (50%) mounted *de novo*, high-magnitude neoantigen-specific T-cell responses by ex vivo IFNγ ELISpot. Using CloneTrack TCR tracking, vaccine-expanded clones constituted up to 10% of all blood T cells, re-expanded after a booster, and persisted (with durable polyfunctional CD8^+^ effector features) despite chemotherapy. At 18-month median follow-up, patients with vaccine-expanded T cells had longer recurrence-free survival (median not reached) versus non-responders (13.4 months; P = 0.003) (84). In one case, all vaccine-expanded clones infiltrated a transient liver lesion consistent with a micrometastasis that subsequently regressed, illustrating potential *in vivo* anti-tumor activity (84). These data support the feasibility of personalized mRNA neoantigen vaccination to induce robust, trackable T-cell immunity and underscore the value of TCR repertoire tracking as a pharmacodynamic readout.

### 10.4 Biologic drug development: minimizing immunogenicity

For widely used biologics, immunogenicity is often restricted by HLA class II. In the prospective, multicenter PANTS cohort, carriers of HLA-DQA1*05 nearly doubled the risk of developing anti-drug antibodies (ADA) against infliximab or adalimumab, independent of treatment regimen. Among infliximab monotherapy patients carrying this HLA, immunogenicity reached 92% in one year, compared with 10% in HLA-DQA1*05–negative patients receiving adalimumab combination therapy (85). However, this difference may reflect the distinct nature of the two antibodies, chimeric antibody (Infliximab) versus fully human antibody (Adalimumab) (85). To mitigate immunogenicity at the preclinical stage, de-immunization has now become standard practice. Tools such as NetMHCpan-4.1 and NetMHCIIpan-4.0, which integrate binding motifs with mass spectrometry, identify ligands, allow reliable prediction of peptide presentation across diverse HLA backgrounds (86). These platforms support iterative screen/mutate/retest cycles to remove helper T-cell epitopes while preserving protein function, thereby reducing ADA risk and improving candidate selection before costly clinical trials.

## Conclusion and future directions: toward precision medicine

Immunopharmacogenomics is revolutionizing our ability to understand and predict drug responses. By linking immune repertoire diversity to clinical outcomes, it provides unprecedented insight into both therapeutic efficacy and immune-mediated toxicity. Across a broad spectrum of conditions, including drug-induced hypersensitivity in skin and liver, cancer immunotherapy, graft-versus-host disease, food allergy, and solid organ transplantation, high-throughput immune repertoire sequencing has emerged as a powerful biomarker platform. It can capture the dynamic interplay between host genetics, drug exposure, and immune activation, enabling mechanistic understanding and precision-guided intervention or patient selection.

Despite these advances, significant challenges remain. Many HLA–disease or HLA–drug associations are population-specific, highlighting the need for globally representative cohorts. Immune repertoire datasets are enormously complex, demanding sophisticated computational and machine learning approaches to extract clinically actionable/applicable insights. Moreover, achieving robust predictive models will require integration with complementary modalities, including transcriptomics, proteomics, metabolomics, microbiome profiling and circulating biomarkers. These multi-dimensional strategies should promise to transform immunopharmacogenomics from a research tool into a cornerstone of personalized medicine. The translational promise of immunopharmacogenomics lies in early risk prediction, personalized therapy optimization, and the rational design of next-generation vaccines and biologics. Ultimately, embedding immunopharmacogenomics into routine clinical practice could shift medicine from population-level prescribing toward truly individualized treatment including immunotherapy, extending survival, preventing severe adverse events, and delivering safer, more effective treatments across diverse patient populations.

Lastly, the future of Immunopharmacogenomics fundamentally depends on the establishment of international collaboration. Only through coordinated, cross-border efforts, scientists can assemble the genomic and immunological breadth necessary to capture the full diversity of global populations. Therefore, a primary recommendation is to establish international data-sharing frameworks that integrate immune repertoire profiles, HLA genotypes, drug-response and disease phenotypes, and clinical outcomes into standardized, interoperable repositories governed by transparent ethical and privacy guidelines. Such frameworks would overcome the current fragmentation of small datasets and enable researchers worldwide to compare immune responses across different ethnicities, therapeutic classes, and disease contexts.

Equally critical is the development of harmonized standards for immune and genomic profiling—including protocols for sample preparation, sequencing, data annotation, and reporting—so that datasets generated in different countries can be meaningfully combined, interpreted, and validated. This harmonization will facilitate regulatory recognition of immunopharmacogenomic biomarkers and accelerate their clinical translation. At the same time, future collaborative initiatives must prioritize equitable global participation, ensuring that low- and middle-income countries (LMICs) have access to technical resources, computational infrastructure, and capacity-building programs. Inclusion of these populations not only promotes global fairness but also strengthens the scientific foundation of immunopharmacogenomics by encompassing the full range of human genetic and immunological diversity.

Furthermore, sustainable collaboration should engage academic institutions, pharmaceutical industries, and regulatory agencies working in synergy to translate immunopharmacogenomic discoveries into practical diagnostic tools and personalized treatment strategies. Ultimately, the advancement of immunopharmacogenomics will hinge on the global scientific community’s commitment to share knowledge, align technical and ethical standards, and build inclusive, transparent infrastructures capable of transforming diverse genomic data into clinically actionable insights that promote safer, more effective, and equitable immune-based therapies worldwide.
